# The structure-based reaction mechanism of urease, a nickel dependent enzyme: tale of a long debate

**DOI:** 10.1007/s00775-020-01808-w

**Published:** 2020-08-18

**Authors:** Luca Mazzei, Francesco Musiani, Stefano Ciurli

**Affiliations:** Laboratory of Bioinorganic Chemistry, Department of Pharmacy and Biotechnology, University of Bologna, Viale G. Fanin 40, 40127 Bologna, Italy

**Keywords:** Nickel, Urease, Catalytic mechanism, Crystal structure, Sporosarcina pasteurii, Klebsiella aerogenes, Helicobacter pylori

## Abstract

This review is an attempt to retrace the chronicle that starts from the discovery of the role of nickel as the essential metal ion in urease for the enzymatic catalysis of urea, a key step in the biogeochemical cycle of nitrogen on Earth, to the most recent progress in understanding the chemistry of this historical enzyme. Data and facts are presented through the magnifying lenses of the authors, using their best judgment to filter and elaborate on the many facets of the research carried out on this metalloenzyme over the years. The tale is divided in chapters that discuss and describe the results obtained in the subsequent leaps in the knowledge that led from the discovery of a biological role for Ni to the most recent advancements in the comprehension of the relationship between the structure and function of urease. This review is intended not only to focus on the bioinorganic chemistry of this beautiful metal-based catalysis, but also, and maybe primarily, to evoke inspiration and motivation to further explore the realm of bio-based coordination chemistry.

## Introduction

**Scheme 1.**
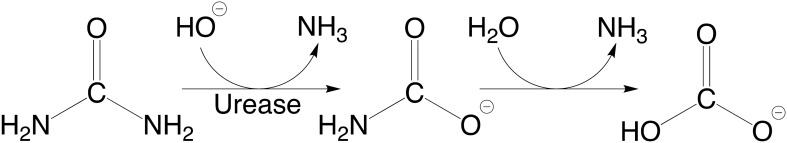
Enzymatic steps for the urea hydrolysis

**Scheme 2.**
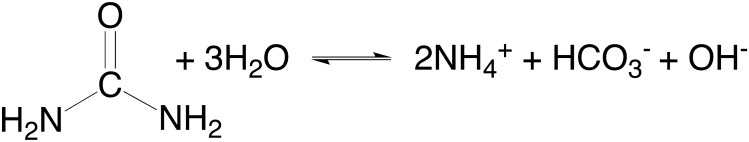
Overall reaction of urea hydrolysis

Urease (urea amidohydrolase E.C. 3.5.1.5) is a nickel-dependent enzyme found in a large variety of organisms, including plants, algae, fungi, and several prokaryotes [1, 2]. It is critically involved in the mineralization step of the global nitrogen cycle, being able to catalyze the rapid hydrolytic decomposition of urea to produce ammonia and carbamate, the latter eventually decomposing spontaneously into a second molecule of ammonia and bicarbonate (Scheme 1) [3–6]. This catalytic activity triggers a rapid overall pH increase of the milieu (Scheme 2) that has negative effects both on human health [7] and agriculture [8].

This alkalization effect is utilized by numerous human pathogenic microorganisms that exploit urease as a virulence factor to infect and colonize the host [7, 9, 10]. The priority pathogen list indicated by the World Health Organization for the research and development of new antibiotics [11] includes urease-dependent antibiotic-resistant bacteria, several of which are involved in bacterial infections of the respiratory apparatus, and it is remarkable that half of patients who died of the recent COVID-19 epidemics in Wuhan (China) became co-infected with bacteria in the lungs and also required antibiotics [12]. In particular, *Helicobacter pylori* infection, affecting large portions of the entire human population, causes a series of gastrointestinal diseases, including chronic gastritis, peptic ulcer and eventually gastric cancer [13]. In *H. pylori*, urease represents up to 10% of the total protein content [14] and is essential for the survival of this human pathogen in the acidic gastric environment by maintaining its cytoplasmic pH close to neutral [15, 16]. In *Staphylococcus aureus*, a human pathogen that causes acute and chronic infections resulting in significant morbidity, urease is crucial to pH homeostasis and viability in urea-rich environments, rendering it an important factor required for persistent murine renal infections [17]. *Mycobacterium tuberculosis*, the etiologic agent of the tuberculosis disease, is an intracellular bacterium that infects macrophages, living inside their phagosomes. In this environment, its survival depends on the activity of nickel-dependent urease. In particular, urea hydrolysis is essential for bacterial survival, since it contributes to nitrogen availability and environmental pH modulation [18]. Moreover, ammonia derived from this reaction can block the phagosome–lysosome fusion, being an important defensive mechanism against the immune system of the host [19]. The alkalizing effect of the urease activity within the mycobacterium-containing vacuole in resting macrophages, and the role for the urease activity in *M. tuberculosis* nitrogen metabolism that could be crucial for the pathogen's survival in nutrient-limited microenvironments where urea is the sole nitrogen source, have been demonstrated [20]. *Yersinia enterocolitica* and *Y. pseudotuberculosis,* isolated from different sources, including food, clinical material and certain animals, can cause acute or chronic foodborne disease manifested by a variety of clinical symptoms, and in Europe yersiniosis is the third most common food-borne gastroenteritis after campylobacteriosis and salmonellosis. Ureases appear to play a vital role in the survival of *Y. enterocolitica* cells in the natural environment by degrading urea in the soil and water, which is utilized by this saprophyte as the sole nitrogen source [21]. Finally, several fungi pathogenic to humans have urease activity, among which is *Cryptococcus neoformans*, whose urease appears to be a component of the composite cryptococcal virulence phenotype, suggesting that urease inhibitors or vaccines may be useful in the treatment or prevention of cryptococcosis [22].

In other settings, the widespread presence of urease in soils, both inside living cells of plants and microbes as well as extracellular enzyme adsorbed onto organic and inorganic soil components, poses significant environmental and economic problems: it causes the release of large amounts of ammonia N in the atmosphere, thus negatively affecting the efficiency of urea-based soil fertilization, inducing plant damage by ammonia toxicity and soil pH increase [8] with the consequent formation of airborne particulate matter (PM) that contributes to atmospheric pollution [23]. It has been found that the presence of ultrafine PM has been significantly associated with an increase of the mortality rate in the SARS (severe acute respiratory syndrome) epidemics in the early 2000s [24], suggesting that containment of air pollution through well-managed agricultural activities is absolutely necessary not only for the environment but also for human health.

The central role of this enzyme in such important aspects of the world society at large has been of great stimulus for the scientific community to extensively investigate and deepen the comprehension of the structure–function relationships of urease, a mandatory prerequisite for the discovery of new chemicals able to challenge its negative effects. The availability of information on the genetic organization of DNA responsible for urease expression, as well as the molecular structures of native ureases and urease-inhibitor complexes, has led to significant steps towards these goals. A substantial improvement in the knowledge of the molecular basis of the catalytic mechanism, including the role of nickel, has allowed us to obtain an essentially complete picture of the enzymatic mechanism. The following paragraphs are a historical excursus on the proposed models for the breakdown of urea catalyzed by urease, the main focus of this monograph.

## Brief history of urease milestones (1864–2010)

The history of urease can be dated back to 1864, when Van Tieghem isolated the first ureolytic microorganism, *Micrococcus ureae* [25]. Five years later Frédéric Alphonse Musculus isolated the first ureolytic enzyme, called “soluble ferment” and able to produce ammonia in putrid urine [26, 27]. In 1926, James B. Sumner, working on the seeds of *Canavalia ensiformis* (jack bean) with the aim of defining the chemical nature of enzymes, obtained isolated protein crystals possessing, using his own words, “to an extraordinary degree the ability to decompose urea into ammonium carbonate”. This property, identical to the already established ability of urease, paved the way for the first demonstration that enzymes were proteins [28]. This discovery led Sumner to receive the Nobel Prize in Chemistry in 1946. During the 1950s to the 1970s, a very fruitful period for the improvement of the knowledge on the structural and biochemical information of enzymes, key aspects as proficiency, stability, and high specificity of urease were established [29].

In 1975, jack bean urease was demonstrated to require nickel for the catalysis, providing the first model for the biological role of this metal as an enzyme prosthetic group [30]. A personal account by Nick Dixon on the discovery of the role of nickel in urease has been published [31].

**Fig. 1:**
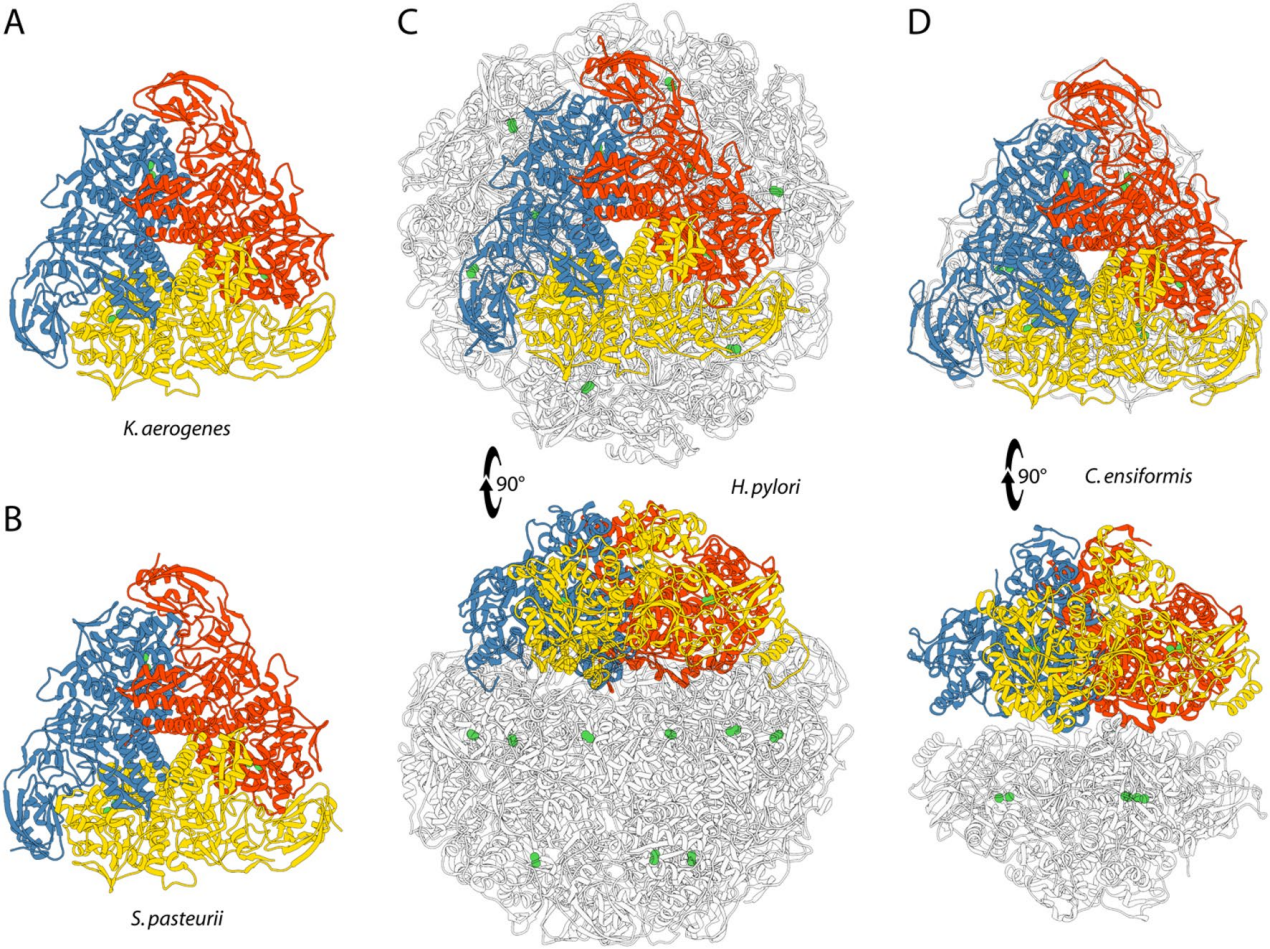
Ribbon diagram of urease from **a** *K. aerogenes* (PDB code: 1EJZ), **b** *S. pasteurii* (PDB code: 4CEU), **c** *H. pylori* (PDB code: 1E9Z), and **d** *C. ensiformis* (jack bean, PDB code: 3LA4). Ribbon colors evidence the chains composing the trimer constituting the minimal quaternary structure of urease. Ni(II) ions are reported as green spheres. The bottom panels of **c** and **d** are rotated by 90° around the horizontal axis vs*.* the top panels

Twenty years later, the first X-ray crystal structure of urease (from the enzyme *Klebsiella aerogenes* (KAU), recombinantly obtained using *Escherichia coli* cell strains) was determined [32]. This important achievement was followed by the determination of the urease structure from the soil bacterium *Sporosarcina pasteurii* (SPU) [33] (formerly known as *Bacillus pasteurii* from older taxonomies) and that of urease from *Helicobacter pylori* (HPU), a human pathogen [34]. The structure of the first plant urease, from *Canavalia ensiformis* (JBU), exactly the same protein that was crystallized by Sumner 85 years earlier, was then reported in 2010 (Fig. 1) [35].

Together, these structures provided a general description of the structure–function relationships of ureases. Nowadays it has been well-established that the overall protein scaffold is conserved among ureases from different sources (Fig. 1). In most bacterial ureases, the quaternary structure is made of a (αβγ)_3_ trimer of trimers with three identical active sites, each located in the α subunits. Some other bacterial enzymes show a larger β subunit resulting from the fusion of the original β and γ subunits, forming (αβ)_3_ trimers. In the special case of urease from *Helicobacter pylori* (HPU), four (αβ)_3_ trimers form the spheroid-shaped tetramer of trimers [(αβ)_3_]_4_, containing twelve independent active sites. Finally, plant ureases are generally made up of a dimer of homotrimers (α_3_)_2_, where the α subunit is derived from the fusion of the corresponding α, β and γ subunits found in bacteria (Fig. 1). The knowledge of the structural properties of the protein architecture did not lead, however, to an immediate general consensus on the reaction mechanism, also because of initial significant differences in the interpretation of the electron density maps derived from X-ray diffraction. These early controversies have now been resolved and what follows is an historic account of the evolution of the consensus on the catalytic steps in the urease mechanism.

## The Australian mechanism (1975–1980)

**Scheme 3.**
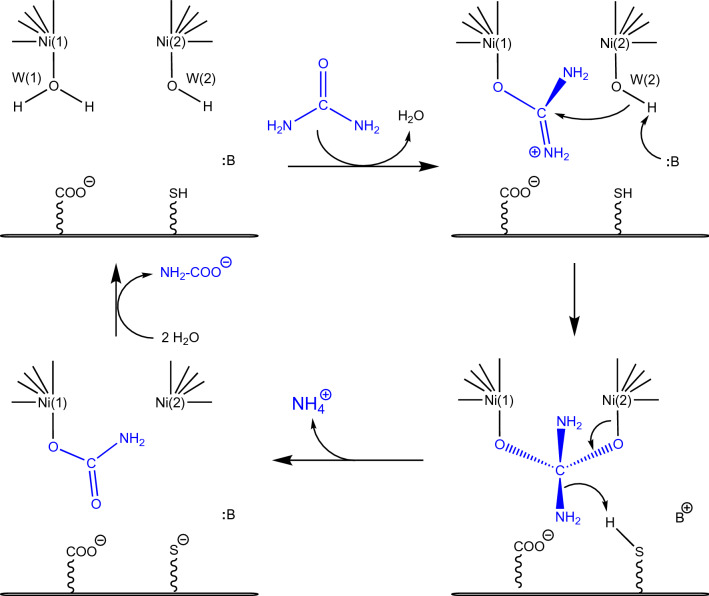
Urease mechanism proposed by Blakeley and Zerner (adapted from ref [4])

In the 1970s, a major discovery in the bioinorganic chemistry field was achieved by Dixon, Blakeley and Zerner, researchers working at the University of Queensland (Australia), who first demonstrated the requirement of two Ni atoms per each of the six subunits of JBU to perform its catalytic activity [30]. At that time, there was no any other information available on the overall structure of ureases, nor any model was developed to describe the activation of carboxylic acid amides towards Ni-dependent hydrolysis or, more in general, metal-ion driven hydrolysis of urea. In this pioneering work, the formulation of a mechanistic hypothesis driven by both nickel ions found in the active site (Scheme 3) was elaborated by analyzing the reactivity of different substrates as catalyzed by the enzyme [4].

In particular, the authors proposed that, in the resting state of the enzyme, one of the two active site nickel ions [Ni(1) and Ni(2), hereafter] would coordinate a water molecule [W(1)] and the other a hydroxide ion [W(2)]. The initial step of this mechanism entailed the replacement of W(1) by a urea molecule, bound to Ni(1) in a monodentate mode using its carbonyl oxygen. Urea would be additionally stabilized through the interaction of one of its NH_2_ groups with a nearby negatively charged carboxylate group from aspartate or glutamate residues. The subsequent nucleophilic attack on the urea carbonyl C atom would be carried out by the Ni(2)-coordinated hydroxide, to form a tetrahedral intermediate that would readily collapse to form carbamate, which would remain, at this stage, coordinated to Ni(1) through one of its O atoms. Carbamate was indeed known to be the product of the urease-catalyzed hydrolysis of urea, evidence that excluded the possibility of an elimination mechanism [3]. The concomitant production of an ammonium cation would be facilitated by an active-site thiol group from a nearby cysteine residue [36] acting as a general acid catalyst. In the last step, the resting state of the enzyme would be regenerated by the entrance of water molecules and release of carbamate. Remarkably, the authors state that “[this] detailed mechanism requires that the two nickel ions … be within ~ 6 Å of each other”, a conclusion that will be proven absolutely correct.

## A mechanism based on spectroscopic and kinetic evidence (1980–1996)

In the fifteen years following the Australian hypothesis, the architecture of the active site of the enzyme was investigated using spectroscopic studies. UV–visible absorption spectra were interpreted as indicating the presence of Ni(II) ions in a six-coordinated pseudo-octahedral geometry in the active site of JBU, while the presence of four- and five-coordinated Ni(II) ions was considered unlikely [30, 37, 38]. X-ray absorption spectroscopic (XAS) studies were also interpreted as suggesting the presence of pseudo-octahedral Ni(II) ions coordinated, on average, to three histidine N atoms at 2.04 Å, two O atoms at 2.07 Å, and one O atom at 2.25 Å [39, 40]. Magnetic susceptibility studies using JBU were then interpreted with the presence of a metal cluster containing two high-spin (*S* = 1) octahedrally coordinated Ni(II) ions, with a weak anti-ferromagnetic coupling [41]. This result confirmed the early assumptions of the presence of two closely-spaced Ni(II) ions [4], and was further supported by the diamagnetism observed upon binding of the competitive inhibitor 2-mercaptoethanol to JBU, resulting in a strong anti-ferromagnetically coupled Ni(II)-Ni(II) dimer bridged by a thiolate *S* atom [41], and by the evidence that this binding involved a ligand exchange in the coordination sphere of nickel [42]. This result was challenged by a later study [43]. Subsequent higher quality XAS data on JBU were interpreted with a model involving the presence of Ni(II) ions bound to five or six (N/O) donor ligands at an average distance of 2.06 Å in a distorted octahedral geometry [42], largely confirming the earlier study [39, 40]. This conclusion was further refined using evidence based again on XAS, which suggested the presence, both in JBU and in KAU, of two penta-coordinated Ni(II) ions in a Ni(N-His)_*x*_(N/O)_5-*x*_ (*x* = 2 or 3) ligand environment, separated by 3.26 Å in the presence of 2-mercaptoethanol, assumed to bridge the two metal ions through the thiolate S atom [44]. Shortly after, an XAS study on SPU was interpreted as indicating the presence, in the enzyme active site, of two hexa-coordinated Ni(II) ions with a Ni(N-His)_2_(N/O)_4_ (*x* = 2 or 3) pseudo-octahedral geometry and an average nickel-ligand distance of 2.03 Å [45]. These preliminary structural information were complemented by kinetics studies on KAU: the pH-dependent activity of the native enzyme, which followed a bell-shaped curve, was shown to be altered in the case of chemical modifications and mutants of αCys319 [46, 47] and αHis320 [48]; these observations were interpreted with the assumption of a role of a general acid for αCys319, consistently with the Australian hypothesis [4], and of a general base for αHis320, having a p*K*_a_ around 6.5 and thus being deprotonated at the optimal pH for catalysis (7.5–8.0). The latter residue was thus proposed to activate a nickel-bound water molecule, yielding the hydroxide ion responsible for the nucleophilic attack on urea during catalysis [48].

## The crystal structures and the American mechanisms (1995–1997)

**Fig. 2:**
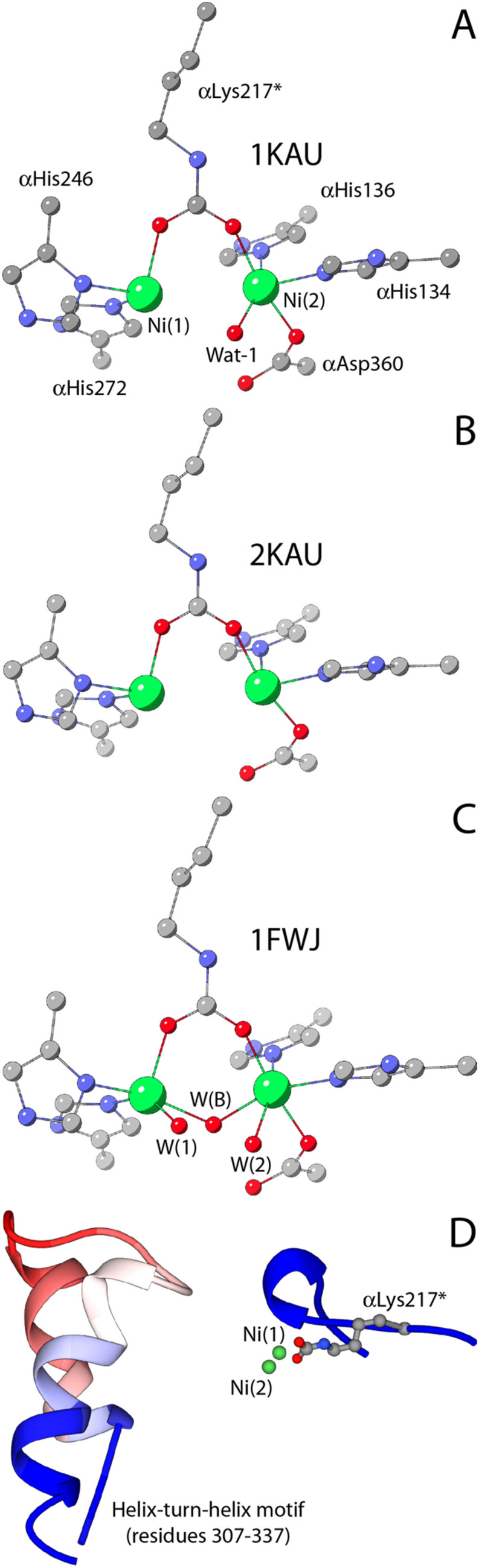
Model structures of the active site of *K. aerogenes* urease (KAU) as evolved from the initial X-ray diffraction data (**a** PDB code 1KAU; **b** PDB code 2KAU) to the more recent interpretation (**c** PDB code 1FWJ). **d** Displays the ribbon diagram of the active site of KAU, highlighting the mobile flap in the closed conformation depicted according to the B-factor, thus showing the large mobility in this key feature of urease

In 1995 the first two X-ray crystal structures of native urease were reported from the bacterium *Klebsiella aerogenes*, one with the PDB code 1KAU, and the other with the PDB code 2KAU (Fig. 2a,b) [32]. In both cases, KAU was shown to oligomerize as a trimer of trimers in a (αβγ)_3_ triangular arrangement (Fig. 1). In the structures, three active sites were identified, located in each α subunit and containing two closely spaced Ni atoms [defined as Ni(1) and Ni(2)] separated by 3.5 Å, in accordance with previous spectroscopic results [44] (Fig. 2). The presence of a carbamylated lysine residue, indicated as αLys217*, bridging the two Ni atoms using its Oθ1 and Oθ2 atoms, was observed, consistently with carbon dioxide requirement for the in vitro activation of urease [49]. Other common features for the two structures were the coordination of Ni(1) to αHis246 Nδ, αHis272 Nε, and the Oθ1 atom of αLys217*, and the binding of αHis134 Nε, αHis136 Nε, αAsp360 Oδ1, and the Oθ2 atom of αLys217* to Ni(2) (Fig. 2). In both structures, Ni(1) resulted in an unprecedented three-coordinated geometry. In the 1KAU structure, a water molecule (indicated as Wat-1) was reported to bind Ni(2) to complete a distorted bipyramidal penta-coordination geometry (Fig. 2a). The authors further affirmed that, in addition to Wat-1, a water molecule weakly binds Ni(1), thus completing a pseudo-tetrahedral coordination geometry; however, this water molecule was not refined in the model and it is not present in the structure deposited in the PDB. Additionally, a suggestion was made that Wat-1, even though refined at full occupancy as a terminal ligand to Ni(2), could also be moving onto two additional positions, either as terminal ligand for Ni(1) or as bridging the two Ni atoms [32]. On the other hand, no nickel-bound water molecule is found in the 2KAU refined model structure, which thus features a tetra-coordinated Ni(2) with an atypical geometric arrangement (Fig. 2b). In the paper, however, the authors stated that, in the case of the X-ray diffraction dataset that eventually yielded 2KAU, excess electron density was detected, having the appropriate size for a urea molecule or a bicarbonate ion, but it was not refined into a model [32].

The coordination geometries that emerged from these first two crystal structures were not, in any case, consistent with the previous XAS studies reported by the same group on KAU, which were interpreted with the presence of two penta-coordinated Ni(II) ions [44]. These inconsistencies were partially resolved later on, when a new crystal structure of KAU was made available by the same authors (PDB code 1FWJ) [50]. In that study, the electron density around the Ni(II) ions was interpreted as due to the presence of three water molecules: W(1) bound to Ni(1), W(2) bound to Ni(2) and W(B) in a nickel-bridging position (Fig. 2c). However, the O⋯O distances between these three Ni-bound water molecules (2.0–2.5 Å) were considered too short to allow them to be simultaneously present, and were refined with variable occupancy [0.79, 1.25 and 0.90 for W(1), W(2) and W(B), respectively]. In this way, Ni(1) appeared to be penta-coordinated in a distorted square-pyramidal geometry, while Ni(2) was hexa-coordinated in a pseudo-octahedral ligand environment, in agreement with all previous spectroscopic observations [50]. However, the authors stated that “the high occupancy of these three water positions that can only be partially occupied suggests that this interpretation is not the complete story” [50]. This was a critical point, as the solvation state of the active site of this hydrolytic enzyme is a key information required to understand its mechanism.

The active site of KAU was found in a pocket enclosed by αAla167, αHis219, αGlu220, αAsp221, αGly277, αCys319, αHis320, αArg336, αAla363, and αMet364. Residues αCys319 and αHis320 were also found to be part of a 30-residues helix-turn-helix (from residues 310 to 339) covering the active site cavity, that was described as highly mobile and suggested to change its conformation from a closed state, as found in the 1KAU, 2KAU and 1FWJ, to an open state (not observed) in order to allow urea extensive access to the active site (Fig. 2d) [32].

**Scheme 4.**
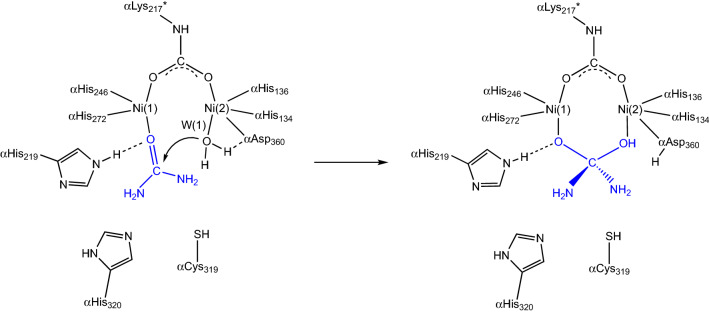
First proposal for the urease reaction mechanism by Hausinger et al. [32]

Guided by the structural information on KAU, Hausinger et al. proposed a mechanism that involved different roles for the two Ni(II) ions [32]. In the first step of this hypothesis, urea would bind in a mono-dentate mode in the active site of urease by coordinating to the least coordinatively saturated Ni(1) via its carbonyl oxygen atom, completing a tetra-coordination environment for this ion and causing polarization of the carbonyl group, consistently with the Australian mechanism [4]. The structure of KAU further suggested that this interaction is aided by αHis219 NεH acting as a H-bonding donor to the urea O atom, corroborating previous functional studies [48], and that the carboxylic groups in the side chains of αGlu220, αAsp221 and the Ni(2)-binding αAsp360, as well as the backbone carbonyl O atoms of αAla167, αGly277 and αAla363 could favor the binding of urea by providing H-bond acceptors for the substrate amide group (Scheme 4) [32].

In the second step, a hydroxide ion, identified as the Wat-1 solvent molecule bound to Ni(2) in the 1KAU structure, was proposed to act as the nucleophile for the reaction by attacking the carbonyl C atom of urea with the formation of a tetrahedral intermediate (Scheme 4). In the final step, the tetrahedral intermediate was assumed to decompose, with the participation of an unidentified generic acid that was previously proposed to protonate a C_urea_-NH_2_ group [51]. This mechanism modified the previously suggested roles for αCys319 and αHis320 [46–48]. In particular, the structure of KAU excluded the possibility for αCys319, preliminary classified as the general acid [46, 47], to carry out this role because it is too far from the position of the urea amide group that needs to be protonated, and was instead implied in assisting the correct orientation of the adjacent αHis320 residue by steric effects [32]. On the same basis, the structure of KAU excluded that αHis320 could act as the catalytic base needed to generate the nucleophilic hydroxide, as initially hypothesized [48], because of the large distance from Wat-1 [32]. The action of a general base was instead suggested, on the basis of the KAU structure, to be carried out by the Ni(2)-bound αAsp360 Oδ1 atom [32].

**Scheme 5.**
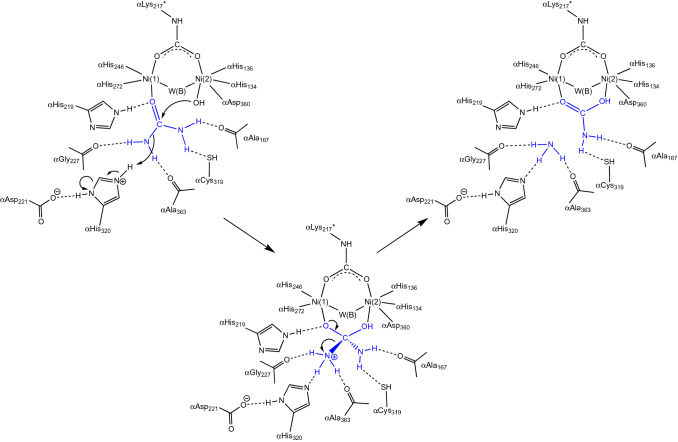
Second proposal for the urease reaction mechanism by Hausinger et al. [29, 50]

The new structural framework for KAU, obtained in 1997 and involving three solvent molecules around the dinickel cluster [50], suggested a further modification to the initial hypothesis depicted in Scheme 4 [29, 50]. According to this revised mechanism, urea would bind Ni(1) with its carbonyl O atom, an interaction stabilized by H-bonds not only involving αHis219 NεH as initially suggested, but also comprising four additional H-bonds between the amide hydrogens of urea and αGly277, αAla363, αAla167 carbonyl O atoms as well as, possibly, αCys319 Sγ acting as an H-bond acceptor (Scheme 5). Similar to their first proposal, in this revised mechanism Hausinger et al. suggested that the subsequent step would be the nucleophilic attack by the hydroxide form of the Ni(2)-bound water molecule onto the urea C atom, to form a tetrahedral hydrated urea intermediate. The latter would witness a large increase in the basicity of the amide N atoms, facilitating the subsequent transfer of a proton from the protonated form of the side chain imidazole of αHis320, in a step that would occur concomitantly or after the nucleophilic attack. As in the first proposed mechanistic model, the protonated tetrahedral intermediate would quickly collapse upon formation of the C_urea_-NH_3_^+^ group to yield ammonia and carbamate, which would subsequently escape from the active site restoring the enzyme native form in a non-rate-limiting step.

The substantial modification proposed for the role of αHis320 with respect to the previous hypothesis, assumed that this residue undertakes a dual role in (i) aiding the maintenance of a correct active site geometry that allows productive urea binding, and (ii) acting as a general acid able to produce a good leaving group during urea breakdown. This hypothesis was inconsistent with previous observation by the same group that suggested that αHis320 is deprotonated at the optimal pH for catalysis (7.5–8.0) [48]. Indeed, to function as a general acid, αHis320 should be protonated, which raised the dilemma of how an enzyme with a pH optimum near 8 could require the protonated form of a group with p*K*_a_ near 6.5. This conundrum was explained using the so-called “reverse protonation hypothesis” [52, 53], by which the bell-shaped pH profile of urease would result from a low-p*K*_a_ group that must be protonated, and a high-p*K*_a_ group that must be deprotonated, at the pH optimum. For urease, this would mean that αHis320 must be protonated for catalysis and another group with a p*K*_a_ near 9 must be deprotonated. The authors also speculated that the high-p*K*_a_ group could be W(2) itself, because such a p*K*_a_ appeared to be reasonable for a Ni(II)-bound water [54, 55], simplifying the model by ruling out the necessity of a general base able to turn W(2) into a hydroxide ion.

Based on this mechanistic hypothesis, additional considerations were made that concerned the 30-residue, highly mobile, helix-turn-helix motif covering the active site cavity and suggested to regulate substrate access by changing its conformation from a closed to an open state [29, 32]. This hypothesis was necessary not only in order to position αHis320 for proton transfer to the urea amide N, but also to explain enzyme inhibition by chemicals targeting αCys319 [46] as a simple physical effect, namely by not allowing the flap to close properly. However, the open conformation of the KAU mobile flap in the native enzyme remained elusive, with the exception of the structure of the Cys-to-Tyr mutant in which the flap was considered to stay open by steric hindrance [50].

## The crystal structure of urease-inhibitor complexes and the European mechanism (1999–2004)

**Fig. 3:**
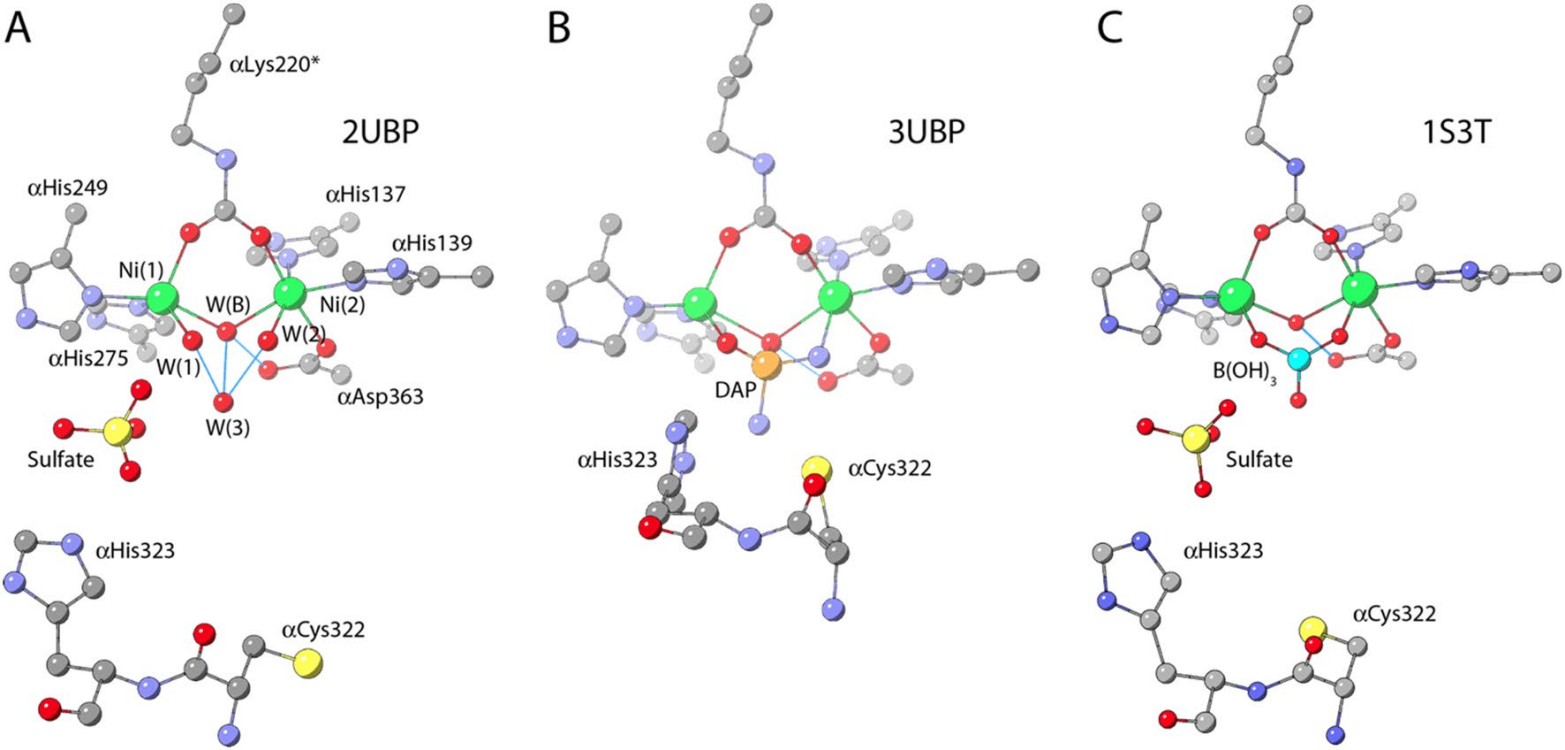
Model structures of the active site of *S. pasteurii* urease (SPU) as derived from X-ray diffraction data in the native state (**a** PDB code 2UBP), bound to diamidophosphate, DAP (**b** PDB code 3UBP) and to boric acid (**c** PDB code 1S3T)

Shortly after the structural characterization of KAU, in 1999 the crystal structure of *Sporosarcina pasteurii* (formerly known as *Bacillus pasteurii*) urease (SPU) [33] was determined (PDB code 2UBP). The overall tertiary and quaternary structures of SPU were found to be very similar to that of KAU (Fig. 1). The coordination environment of the Ni(II) ions in the active site of SPU was also largely comparable to that of KAU (Fig. 3a). In particular, the two Ni(II) ions are separated by 3.7 Å and bridged by the Oθ1 and Oθ2 atoms of a carbamylated αLys220* (SPU numbering),^1^ Ni(1) is additionally bound to αHis249 Nδ and αHis275 Nε, while Ni(2) is bound to αHis137 Nδ, to αHis139 Nε, and to αAsp363 Oδ1. The hydration environment of the active site of SPU was clearly described with four well-ordered solvent molecules, W(1), W(2), W(3), and W(B): the latter symmetrically bridges the two nickel ions, whereas W(1) and W(2) complete a distorted square-pyramidal and a distorted octahedral coordination for Ni(1) and Ni(2), respectively. The fourth water molecule, W(3) in a distal position, is at H-bonding distance from W(B), W(1) and W(2). The authors also assigned the protonation state of W(1) and W(2) as neutral water molecules, while W(B) was considered to be a hydroxide ion according to the estimated p*K*_a_ values for a water molecule bound to the Ni(II) hexa-aquo ion (p*K*_a_ around 10.6) and for water-bridged bimetallic complexes (having a very acidic p*K*_a1_ and a p*K*_a2_ slightly lower than the p*K*_a_ of the first ionization of a single ion bound to water) [54]. In this context, the third lone pair of the W(B) hydroxide, not involved in Ni-coordination, was considered to be involved in a H-bond with αAsp363 Oδ2. The H-bonding network of the three Ni-bound water molecules is completed by W(1) receiving a H-bond from αHis222 Nε, which is, as in KAU, protonated as deduced from the interaction of αHis222 Nδ with the peptide NH group of αAsp224, whereas W(2) forms a strong hydrogen bond with αAla170 O, which acts as H-bonding acceptor.

In the case of SPU, the authors provided a rationale for the existence of this pseudo-tetrahedral arrangement of closed-spaced solvent molecules (O⋯O distances in the 2.1–2.4 Å range) around the di-nickel cluster [33], suggesting the presence of a four-centered hydrogen-bonding network involving a proton located in the center of the tetrahedron constituted by W(1), W(2), W(3) and W(B) and assisted by residues acting as H-bonding donors and acceptors (αAsp363 Oδ2, αHis222 Nε, and αAla170 O). In addition, the presence of a sulfate ion close to the di-metallic Ni cluster (Fig. 3a) was proposed to additionally stabilize W(3): this anion forms a hydrogen bond with αHis323 Nε and is located between the four-water/hydroxide cluster and the nearby αArg339, forming a strong salt bridge. While the presence of sulfate in crystals of native SPU is probably due to its high concentration in the crystallization buffer, in native KAU, also obtained from sulfate-rich solutions, its position is occupied by the imidazole ring of αHis320 (αHis323 according to SPU numbering) and none of the published KAU structures shows sulfate binding [29, 32, 50]. W(3) is also at the center of additional possible multiple H-bonding interactions with αAsp363 Oδ2 and αGly280. In addition to the H-bonding network thus described that can rationalize the short O⋯O distances found in the tetrahedral solvent cluster in the active site of SPU, a detailed analysis of the interactions between W(1) or W(2) and the amino acids facing the active-site cavity revealed the presence of close van der Waals contacts between W(1) and αHis249 Cε, αHis249 Nδ, αGly280 O, and αLys220* Oθ1, and between W(2) and αHis139 Cε, αHis139 Nε, αAla366 Cβ and αLys220* Oθ2, which were also used to rationalize the existence of this water “droplet” in the active site of SPU [33]. The structure of native SPU was an unprecedented example of a native urease structure showing the mobile flap covering the active site in the elusive open conformation.

Concomitantly with the crystal structure of native SPU, Benini et al. also determined the crystal structure of SPU bound to diamidophosphate (DAP), a molecule resulting from the enzymatic hydrolysis of phenylphosphodiamidate (PPD) and considered to behave like an analog of the transition state or the intermediate of the urea hydrolysis reaction [33]. In that structure (PDB code 3UBP), the tetrahedral molecule of DAP exactly replaces the cluster of four solvent molecules found in native SPU, binding to Ni(1) and to Ni(2) with one O and one N atom, respectively, with the second O atom bridging the two Ni atoms and the second N atom pointing towards the active site cavity (Fig. 3b). A hydrogen-bonding network similar to that found in the native form of the enzyme stabilizes the ligand in the active site, with the Ni(1)-bound DAP O atom receiving a H-bond from the protonated αHis222 Nε, the Ni(2)-bound DAP N atom donating two hydrogen bonds to the backbone carbonyl O atoms of αAla170 and αAla366, and the Ni-bridging DAP O atom being at H-bonding distance to αAsp363 Oδ2, implying the presence of a proton on the bridging DAP O atom. Finally, the distal DAP N atom donates a bifurcated H-bond to the αAla366 backbone O atom and to Nε atom of the mobile-flap αHis323 residue. The position of αCys322 and αHis323 was significantly shifted as compared to the resting state as a consequence of a large change occurring in the helix-turn-helix region (Fig. 3a, b) that adopts a closed conformation. In this way it was experimentally proven, for the first time, that the urease active site flap could assume two different states–open, as in native SPU, or closed, as in the DAP-inhibited SPU [33] and in all previous native KAU structures, strongly supporting the hypothesis previously suggested by Hausinger et al. that this conformational change is important for the urease mechanism [29].

**Scheme 6.**
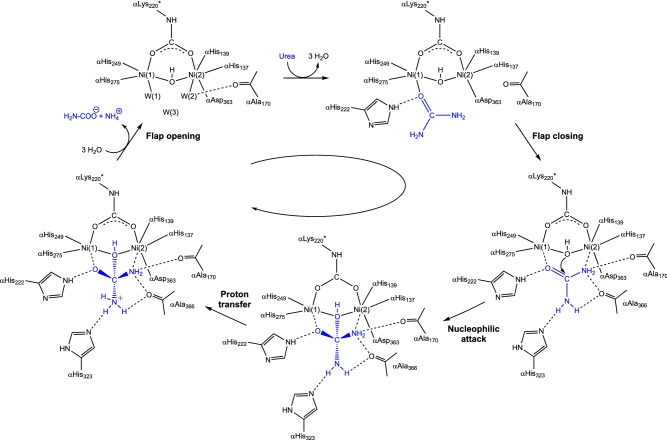
Urease reaction mechanism proposed by Benini et al*.* [33]

Inspired by their findings, Benini et al. proposed an alternative reaction pathway, illustrated in Scheme 6 and referred here as the “bridging hydroxide mechanism” [33, 56]. According to this hypothesis, urea would enter the active site cavity when the flap is in the open conformation, replacing W(1), W(2), and W(3), with αHis222 being involved, as previously demonstrated, in a hydrogen-bonding network that orientates the substrate in the catalytic cavity and stabilizes the initial binding of the carbonyl oxygen to the more electrophilic five-coordinated Ni(1). In this binding mode, one of its amide groups would move close to the six-coordinated Ni(2) and eventually binds, chelating the two metal ions. In the bidentate nickel-binding mode of urea, both Ni(II) ions would have a direct role in substrate binding and activation. This binding mode would be further stabilized by a rearrangement of αAla366 backbone, which tilts its carbonyl O atom towards Ni(2) and, together with the carbonyl group of αAla170, acting as hydrogen-bond acceptors with the urea NH_2_ group. The latter two residues, αAla170 and αAla366, would also assist urea binding to Ni(2) by enhancing the Lewis basicity of the amide group of urea. The orientation of the substrate would be further induced by the asymmetric structural features of the active-site residues, positioned to act as hydrogen-bond donors in the vicinity of Ni(1) and as hydrogen-bond acceptors in the vicinity of Ni(2). These results suggested an enzyme active site specifically designed to selectively bind the substrate in an orientation-specific mode.

Altogether, these interactions polarize the C–O and the C–NH_2_ bonds, bringing the C atom of urea in close proximity to the nickel-bridging hydroxide. In this hypothesis, the latter anion would be the nucleophile that attacks the carbonyl C of urea, forming a tetrahedral transition state. At this stage, a conformational change of the flap from an open to a closed state would ensue. In this scenario, αHis323 in its neutral form would move ca. 5 Å closer to the reaction site and would be in the proper position to form a H-bond between its Nε imidazole atom and the distal -NH_2_ group of the transition state/intermediate of the reaction. At this stage, the proton needed to generate a nascent ammonia molecule through the cleavage of a C–NH_3_^+^ bond could be provided by the bridging hydroxide itself, which is now part of a diamino-(hydroxy)methanolate moiety, and therefore very acidic after the formation of the C–O bond. This event was proposed to occur via the nearby carboxylate group of αAsp363, later shown to undergo a dihedral rotation along the Cα–Cβ bond, approaching alternatively the bridging hydroxide or the distal NH_2_ group, following the determination of the crystal structure of the SPU complex with the inhibitor acetohydroxamic acid [57]. According to this hypothesis, the release of ammonia from the active site would be assisted by the movement of αHis323 when the mobile flap opens. The resulting negatively charged carbamate is then released, owing to the unfavorable interaction between the deprotonated nickel-bridging carbamate oxygen and αAsp363 Oδ2, in a process assisted by the movement of the positively charged αArg339 upon flap opening. According to this proposed mechanism, the bridging hydroxide simultaneously acts as the nucleophile and the general acid, while αHis323 acts by stabilizing the positive charge which develops on the transition state rather than deprotonating the hydrolytic water, as proposed by Hausinger and co-workers in their first hypothesis.

The “bridging hydroxide mechanism” raised some initial criticism among the bioinorganic community during the XXXIII ICCC in Florence (August 1998), the 5th ISABC in Corfu (April 1999), and the 9th ICBIC in Minneapolis (August1999), with negative comments based mainly on the supposed kinetic inertia of a doubly coordinated nucleophile [58]. However, shortly after this hypothesis was proposed, two additional studies were reported that seemed to support it. Specifically, one study reported and discussed the crystal structure of the SPU in complex with phosphate (PHO, PDB code 1IE7), a competitive inhibitor [58], and another described a computational approach to the enzyme mechanism [59]. In particular, the latter confirmed the hypothesis that urea must first bind to the enzyme active site with the flap in the open conformation, as the entrance to the active site would be otherwise prevented by steric clashes. Additionally, the calculations supported the initial binding of the carbonyl O of urea to Ni(1), displacing W(1), W(2) and W(3) and leaving the bridging hydroxide in place. However, the binding of one of the amido-NH_2_ groups of urea to Ni(2) was proven not to be favored unless the flap moves into a closed conformation, a phenomenon that decreases the active site volume, forcing this interaction to take place [59]. Closure of the flap would also be responsible for the stabilization of the catalytic transition state through the formation of multiple H-bonds with the active site residues αAla170 and αAla366, as initially proposed. These interactions would induce a change of this N atom from a “pseudo” sp^2^ hybridization, with some sp^3^ character, to a pure sp^3^ hybrid, thus favoring its coordination to Ni(2), which would compensate the loss of resonance delocalization energy of the urea molecule [59]. The same calculations suggested that the nucleophilic attack of the bridging hydroxide occurs concomitantly with the formation of the coordination bond between the urea -NH_2_ group and Ni(2) [59], instead of following it, as originally proposed [33, 56].

In 2004, the determination of the crystal structure of SPU bound to boric acid, B(OH)_3_, considered to behave like a substrate analog [60], reinforced the bridging hydroxide mechanism (PDB code 1S3T). In the SPU-B(OH)_3_ complex, the urea-like inhibitor replaces W(1), W(2) and W(3) by symmetrically coordinating the Ni(II) ions with two oxygen atoms, while the third O atom points toward the cavity opening, not perturbing the position of the bridging hydroxide (WB). In this structure, the H-bond network around the boric acid O atoms resembles that of the water molecules in the native urease (Fig. 3c). The structure revealed that the bridging hydroxide is placed almost perpendicular to the plane of the B(OH)_3_ molecule, with a B⋯OH distance of only 2.1 Å. The different reactivity between urea (a substrate) and boric acid (an unreactive substrate analog and an enzyme inhibitor) was ascribed to unfavorable symmetry and energy of the highest energy orbital carrying the two electrons necessary for the bond formation (the HOMO) on the bridging hydroxide nucleophile and the lowest energy empty orbital (the LUMO) on the inhibitor.

## And yet it moves: the role of the active site flap (1995–2019)

The helix-turn-helix motif covering the active site cavity carries, at the tip of the mobile turn region between the two helices, the fully conserved αHis320/αHis323 residue (KAU/SPU numbering), which has been, in the differently proposed mechanisms described above, assumed to act as a general acid or a general base, but in any case, involved in key catalytic proton transfer steps. The ability of this motif to move, initially suggested by Hausinger et al. for KAU [29, 32] was proven for SPU by comparing the structures of native and DAP-inhibited enzyme [33], which additionally revealed that the position of this key residue shifts by ca. 5 Å towards the di-nickel center upon transition of the flap from an open to a closed conformation. The mobile flap was found in the open conformation in the case of native SPU, as well as in the structures of SPU bound to competitive and uncompetitive inhibitors [5, 6, 33, 56–58, 60–68]. In some of these cases the flap is forced to stay open, as for urease inactivated by 1,4-benzoquinone [64], catechol [66], and heavy metal ions such as Ag(I) [67] and Au(I) [68], supporting the idea that flap closure is absolutely necessary for catalysis to occur.

The structural and functional information available on ureases from different sources thus provided a strong correlation between the efficiency of the catalytic mechanism of urease and the viability for the existence of at least two different conformations for the mobile flap, open or closed. The critical role of the flap in stabilizing the substrate or the transition state into the active site, was initially based on the fact that the structure of native SPU [33], as well as boric acid-SPU complex (an unreactive substrate analog), showed a flap in the open conformation [60], while the structure of SPU bound to the transition state analog DAP, resulting from the in situ hydrolysis of PPD showed a flap in the closed state [33]. This observation was later confirmed in the case of SPU bound to *N*-mono-amino thiophosphate (MATP, PDB code 5OL4), the latter resulting from the enzymatic hydrolysis of *N*-(*n*-butyl)-thiophosphoric triamide (NBPT), and differing from the structure of the DAP-SPU complex only by substitution of the distal N atom of DAP with a S atom in the case of MATP [65]. Altogether, this information reinforced the idea that this structural change is mandatory for urease to stabilize the substrate or the transition state formed upon nucleophilic attack of the bridging hydroxide on urea.

Some reasons for the conformational variability of the mobile flap had been already considered in the early days of urease structural investigations. In particular, two hypotheses were proposed: (i) one by which the extended interactions involved in flap closure favor the open or disordered conformation, so that only favorable interactions caused by the presence of urea in the active site are required to close the flap, and (ii) another that implied that the extended interactions made by the flap are sufficient to favor flap closure, but side chains and water in the urea binding pocket make unfavorable interactions that destabilize the closed state of the empty enzyme [29]. The authors favored the second explanation, considered to be more consistent with the view that the enzyme is designed for maximally effective binding of the transition state [29].

**Fig. 4:**
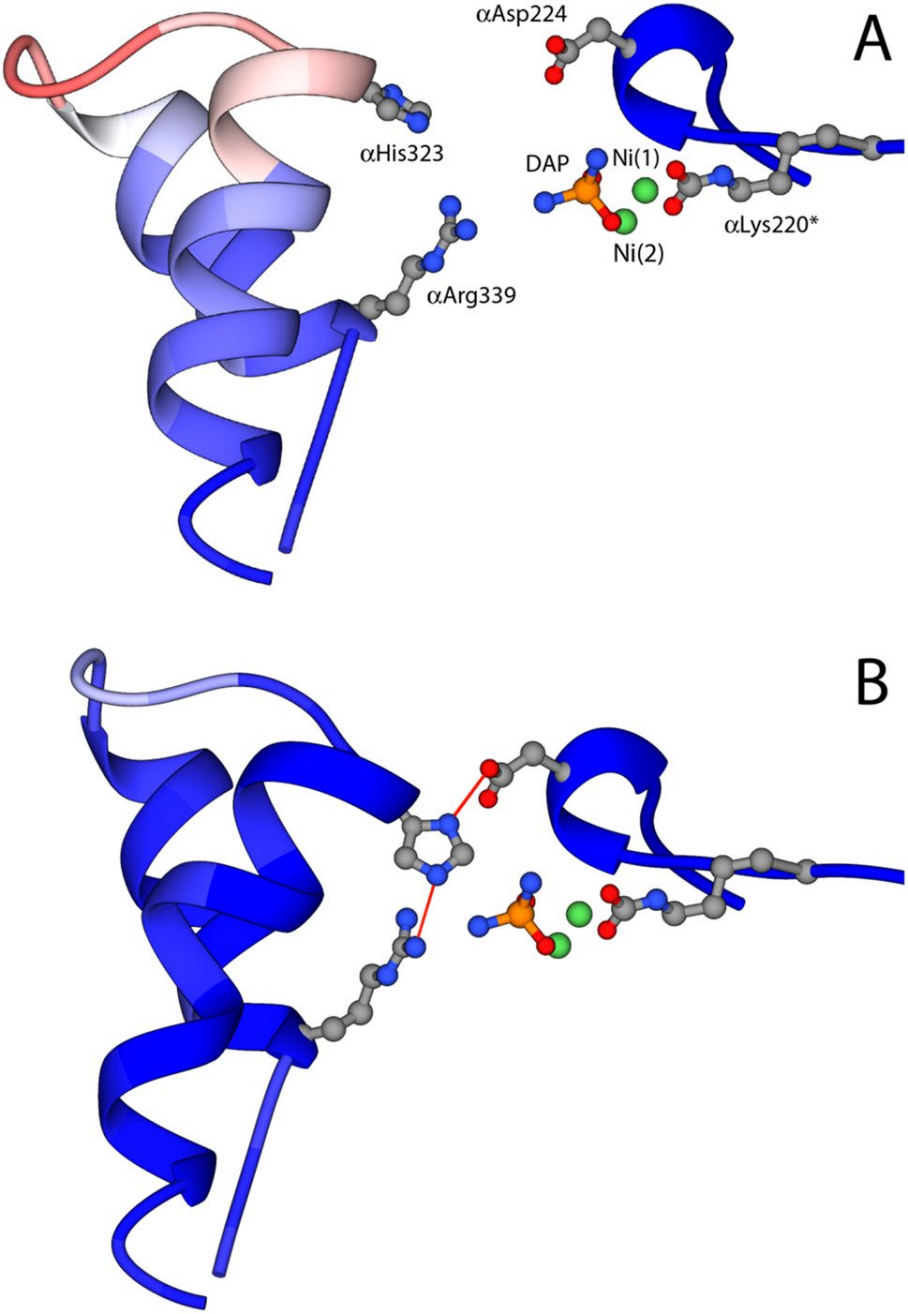
Ribbon diagram showing the active-site flap of SPU inhibited in the presence of NBPTO and bound to DAP in the open conformation at pH 6.5 (**a** PDB code 6RP1) and in the closed conformation at pH 7.5 (**b** PDB code 6RKG). The ribbons are colored according to the crystallographic B-factor. The side chains of αLys220*, αCys322, and αHis323 as well as the two Ni atoms and the bound DAP molecule are also shown

An alternative explanation for the differences between the case of KAU (flap mainly closed) and SPU (flap mainly open) has been recently provided by kinetic and crystallographic studies carried out on SPU inhibited by *N*-(*n*-butyl)-phosphoric triamide (NBPTO) and yielding DAP-SPU complexes [69, 70]. This rationalization, based on the working hypothesis that the different pH reported for the crystallization of KAU (pH > 7) and SPU (pH < 7) is the source of these dissimilarities, was experimentally challenged by determining the structure of SPU inhibited in the presence of NBPTO and bound to DAP at pH 7.5, 7.0 and 6.5 (PDB codes 6RKG, 6H8J and 6RP1, respectively) [69, 70]. The DAP ligand was shown, by ^31^P NMR, not to change protonation state in this pH range [70]. The result of this analysis showed that the stabilization of the flap in an open/closed state is indeed dictated by pH, promoting an open conformation at more acidic-to-neutral pH (Fig. 4a), and a closed conformation at neutral-to-alkaline pH (Fig. 4b) [69, 70]. In particular, a combination of kinetic and structural studies showed that αHis323 in SPU has a p*K*_a_ of ca. 6.6, so that at higher pH values a large percentage of the protein has the flap in the closed conformation and the imidazole group of this residue is deprotonated and found inserted in the active site cleft, forming two hydrogen bonds with αAsp224 Oδ2, using the Nδ atom, and αArg339 Nδ2, using the Nδ atom (Fig. 4b) [69, 70]. The chemical nature of the carboxylic oxygen of αAsp224 as a H-bond acceptor, together with the one of the guanidinium nitrogen of αArg339 as a H-bond donor, suggested that αHis323 is actually protonated at its Nδ and deprotonated at its Nδ. On the other hand, at pH values lower than 6.6, the percentage of the protein with the *open* conformation of the flap progressively increases, with αHis323 gradually moving, on average, farther away from the di-metallic center. In this latter case, the volume of the active site cavity is occupied by a sulfate ion (that is deprotonated at the crystallization pH), which makes two hydrogen-bond interactions through two O atoms with αArg339 Nη1 and Nη2, while a third O atom is placed at 2.2 Å from αHis323 Nε, indicating that the latter, must be protonated. Overall, this analysis indicated that, whereas αHis323 is neutral and protonated only at its Nδ position at the optimum pH 7.5, the same residue is cationic and doubly protonated at pH 6.5. The importance of the conserved residues αAsp224, αHis323, and αArg339 in the correct positioning of the flap during the catalytic process of urease was already suggested by kinetic studies on KAU [71]. In this view, the triad αAsp224-αHis323-αArg339 forms a three-member lock-and-key system, in which the protonation state of αHis323 dictates the flap movement toward and away from the active site. In particular, at pH values greater than the p*K*_a_ of αHis323, Nε would be deprotonated, allowing αHis323 to interact with both αAsp224 and αArg339. In this state, the mobile flap would be more stable in the closed conformation. On the other hand, the protonation of αHis323 Nε, an event that must occur at pH values below its p*K*_a_ of 6.6, renders this residue unable be clamped between αAsp224 and αArg339 because of the positive charge generated on the imidazole ring, which would prevent its interaction with the positively charged side chain of αArg339.

## The fluoride ion as a new player (2000–2014)

The hypothesis of the bridging solvent-derived moiety acting as the catalytic nucleophile in the enzymatic hydrolysis of urea gained further support after the publication of kinetic data of fluoride inhibition of KAU [72]. These studies were interpreted by excluding that fluoride replaces either or both solvent molecules terminally bound to Ni(1) and Ni(2), this time assumed to be in the neutral H_2_O form [72], thus suggesting that fluoride replaces the Ni-bridging solvent molecule [72]. Additionally, the authors indicated that fluoride inhibition would occur following turnover, alias upon urea binding to Ni(1) and not before, an event that could weaken the bond between the bridging solvent molecule, assumed to be a neutral H_2_O considering the pH-dependence of fluoride inhibition [72]. These considerations led the authors to conclude that the “bridging hydroxide” hypothesis was consistent with their result, with the caveat that αHis320 would need to be protonated to act as the general acid in the reaction, differently from what had been proposed in the European mechanism. Shortly after, kinetic studies on KAU mutants of αHis219, αAsp221, αHis320 and αArg336 by the same group were used to support the involvement of the protonated αHis320 as the general acid, and of a bridging solvent molecule as the nucleophile [71]. A variant of this mechanism was also proposed, in which the nucleophile would be the di-anionic oxide ion (O^2−^) and not a hydroxide ion [71].

**Fig. 5:**
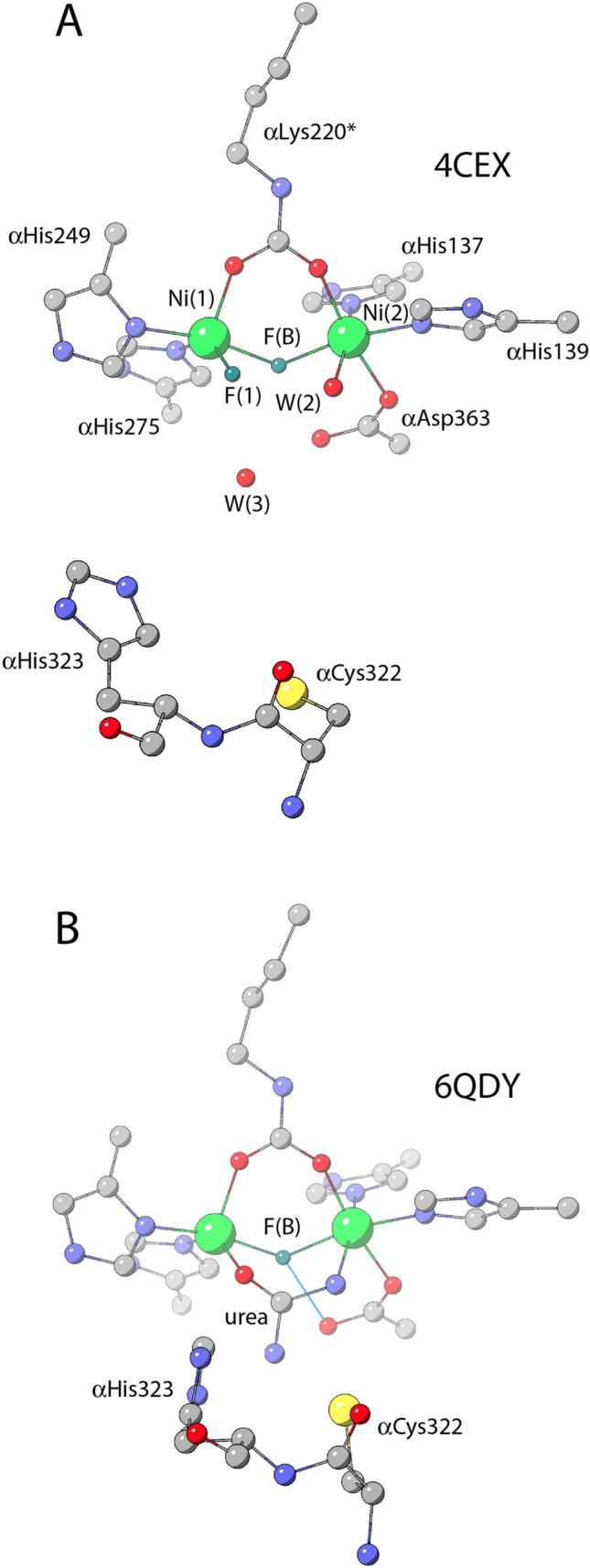
Model structures of the active site of *S. pasteurii* urease (SPU) as derived from X-ray diffraction data in the fluoride-inhibited state (**a** PDB code 4CEX), and in the fluoride-inhibited state bound to urea (**b** PDB code 6QDY)

Additional proof for the “bridging hydroxide” hypothesis was obtained in a study, published in 2014 by Benini and co-workers, reporting a combined kinetic and structural characterization of the inhibition of SPU with fluoride [62]. The study demonstrated that fluoride inhibits SPU with a mixed competitive and uncompetitive mechanism; the latter is predominant and increases with pH increase, while the latter features an opposite pH dependence. The crystal structure of the fluoride-inhibited SPU enzyme (PDB code 4CEX) revealed the presence of two fluoride anions bound to the dinickel cluster (Fig. 5a): one fluoride replaces the bridging solvent molecule, as already suggested by Hausinger and co-workers [72], while an additional fluoride replaces the water molecule terminally bound to Ni(1) [62]. The authors assigned to the anion bound to Ni(1) the competitive role, contending with urea for this site, and to the anion in the Ni-bridging position an uncompetitive role as replacing the co-substrate in the reaction, namely the bridging hydroxide, would require.

## At last, the urease-fluoride-urea complex (2019)

The structural and kinetic information obtained by studying the inhibition of SPU by fluoride suggested experiments aimed at obtaining a ternary complex between SPU, fluoride and urea by co-crystallizing SPU in the presence of urea upon pre-incubation of the enzyme with fluoride (Fig. 5b) [73]. The experiment worked, and the resulting crystal structure (PDB code 6QDY) represents a breakthrough in the understanding of the catalytic mechanism of urea hydrolysis by urease. The structure unambiguously revealed the presence of a fluoride ion positioned in the nickel-bridging position, confirming the previous observations effected through kinetics [62, 71, 72] and structural [62] investigations. Most importantly, the structure revealed the presence of a urea molecule replacing W1, W2 and W3 and binding Ni(1) and Ni(2) in a bidentate mode, using its carbonyl O atom and its amide N atom, respectively. The second amide N atom points away from the Ni(II) ions, towards the active site channel. As postulated by the “bridging hydroxide mechanism”, the urea O atom receives a hydrogen bond from the protonated αHis222 Nε, structurally demonstrating its direct involvement in the formation of the enzyme–substrate complex, while the interaction between Ni(2) and the urea N atom is stabilized by a hydrogen-bond network involving the backbone carbonyl O atoms of αAla170 and αAla366, whereas the carbonyl O atoms of αGly280 and αAla366 stabilize the distal urea N atom through additional H-bonds. This structure clears out any possible doubts associated with this hypothesis, definitely elucidates the coordination mode of urea in the active site cavity, and unambiguously assigns the role of the bridging hydroxide as the nucleophile in the hydrolytic reaction.

In this structure, the mobile flap is observed in the closed conformation, with αCys322 and αHis323 placed in close proximity to the urea molecule in the active site. A network of hydrogen bonds, involving αHis323 Nδ1 and αAsp224 Oδ2, as well as αHis323 NHε2 and αArg339 NHη2, locks αHis323 in the observed position. In this scenario, the described H-bond network imposes that αHis323 must be neutral, supporting the proposition that αHis323 is required to stabilize a nascent ammonia molecule formed upon proton transfer from the bridging hydroxide to the distal amide group of a Ni-bound urea, prior to the breaking of the C–N bond and the release of ammonia. The closing of the flap, which stabilizes the binding of the substrate urea to the Ni ions in the active site, could happen with a frequency that depends on the pH, with an increased probability to find the flap in the closed conformation as the pH increases up to its optimum value of 7.8 [70]. It can be envisioned that, after hydrolysis, the flap swings open allowing release of the products and the entry of a new urea molecule to re-start the catalytic cycle.

## Conclusion

Many years have passed since the discovery of a biological role for nickel in the catalysis of urease, opening a breach in the bioinorganic scientific community through which subsequent studies unearthed an ever-increasing number of nickel-dependent enzymes. This role has been elucidated through a long and winding road, full of surprises, wrong turns and discoveries at each crossroad. We hope and believe that the evolution of the theories concerning the mechanism of this key enzyme, described and discussed in this mini review, could inspire the new generations of bioinorganic chemists to resolve the mysteries still shrouded in the chemistry of the active sites of other metalloenzymes. In the same context, it is interesting to highlight the close relationship of the structure and chemistry of urease with a number of related metallohydrolases [74, 75] such as arginase and agmatinase [76] as well as purple acid phosphatases [77], which feature a similar di-metallic active site containing manganese and iron in place of nickel.

Our contribution is nothing more than an attempt to stimulate the curiosity and interest of our scientific community into the chemical biology of this very ancient element, originated with iron as a major end product of supernova nucleosynthesis, brought to Earth by meteorites, discovered in mid 1700s, given a devilish name, and yet so important for the geo-biochemical cycles of nitrogen, hydrogen, carbon and oxygen, essential elements on which life, as we know it, depends.

## Footnotes

1 In general, the numbering of the amino acid sequence of SPU α subunit in the surroundings of the active site corresponds to that of KAU + 3.
